# Exploring *Castanea sativa* Shells (CSSs) as a Source of AKR1B1 and AKR1B10 Inhibitors: From Extraction to Bioactivity Testing

**DOI:** 10.3390/molecules31030563

**Published:** 2026-02-05

**Authors:** Lucia Piazza, Lorena Tedeschi, Francesca Felice, Antonella Cecchettini, Elisa Ceccherini, Martina Avanatti, Adrian Florentin Suman, Francesco Balestri, Silvia Rocchiccioli, Giovanni Signore

**Affiliations:** 1 Institute of Clinical Physiology, National Research Council, 56124 Pisa, Italy; luciapiazza@cnr.it (L.P.); lorena.tedeschi@cnr.it (L.T.); antonella.cecchettini@unipi.it (A.C.); elisaceccherini@cnr.it (E.C.); adrianflorentinsuman@cnr.it (A.F.S.); giovanni.signore@unipi.it (G.S.); 2 Biochemistry Unit, Department of Biology, University of Pisa, 56123 Pisa, Italy; francesca.felice@unipi.it (F.F.);; 3 Interdepartmental Research Center Nutrafood “Nutraceuticals and Food for Health”, University of Pisa, 56124 Pisa, Italy; 4 Department of Clinical and Experimental Medicine, University of Pisa, 56126 Pisa, Italy

**Keywords:** *Castanea sativa* shells, AKR inhibitors, metabolomics, antioxidants

## Abstract

Chestnut shells are widely recognized as a source of bioactive compounds, including polyphenols and other antioxidant molecules. The industrial chestnut food chain generates large amounts of this by-product, which represents both a waste disposal challenge and a potential source of promising biomolecules. Thermal treatments occurring during industrial processing, however, may affect both chemical composition and bioactivity. Characterization of the chemical composition and biological activity of chestnut shells can contribute to the valorisation of this industrial by-product. Understanding which molecular alterations are caused by the processing is essential to assess the real potential of chestnut shell biomass. This study provides a comparative analysis of *Castanea sativa* shells, both raw and industrially processed. Evaluation was performed at different levels, exploiting mass spectrometry–based metabolite profiling, Total Phenolic Index analysis, antioxidant capacity, and inhibitory activity against AKR1B and AKR1B10, two reductases involved in key physiopathologic pathways. A comparison between extraction solvents (water and ethanol) and processing status (raw versus industrially processed) was performed. Overall, our results support the view that chestnut shell residues represent a valuable source of bioactive extracts. In a circular economy framework, such extracts could be developed to act on AKR1B1/AKR1B10 activity and oxidative stress, thereby contributing to the valorisation of chestnut processing by-products.

## 1. Introduction

Chestnut fruit plays a relevant role in European agriculture. According to FAO statistics, Europe produced 322 K tons of chestnuts in 2023, with Italy among the three top European producers (after Spain and Turkey), contributing 27% of the total. Among the Italian regions, Tuscany ranks fourth in chestnut cultivation, after Campania, Piedmont, and Calabria, with roughly 3000 hectares covered in chestnut trees. Chestnut fruit has long been considered a functional food for its nutraceutical properties, such as the low levels of fat (primarily unsaturated fatty acids) and the high content of antioxidants, including phenolic compounds, vitamin C, and carotenoids [1]. However, behind its beneficial properties, the food manufacturing chain of chestnuts presents some environmental issues. The chestnut fruit shows two protective layers named outer and inner shells (also known as pericarp and integument), accounting for approximately 20% of the total weight, which are discarded during the industrial peeling process [2]. Numerous studies have demonstrated that chestnut shell extracts show antioxidant, antitumor, anti-inflammatory, and anti-adipogenic activities [3,4,5,6]. Chestnut shells are a rich source of polyphenols, among which the most represented are condensed and hydrolysable tannins, including procyanidins made of epicatechin units as well as ellagitannins such as vescalagin and castalagin. Other abundant bioactive molecules identified are phenolic acids, flavonoids, and anthocyanidins [2,5,7]. Recent research has increasingly focused on the valorisation of these industrial by-products characterized by significant biological and functional properties. In fact, the combined effect of industrial processing (usually at temperatures of 200–220 °C for 30–45 min) and extraction conditions on the molecular fingerprint and functional properties of shell-derived extracts is still poorly characterized. Industrial desiccation typically involves rapid thermal treatment at elevated temperatures, which can trigger oxidation, polymerization, or cleavage of labile compounds, especially polyphenols and cinnamoyl-containing structures. Understanding how these processes reshape the extractable compounds is essential to assessing the real potential of chestnut shell biomass within a circular economy framework.

Among the phenolic acids and flavonoids reported in chestnut shell extracts [2,8,9,10], several, such as gallic acid, epigallocatechin, apigenin, luteolin, and quercetin, are known inhibitors of Aldo-Keto Reductase Family 1 Member B1 (AKR1B1) and AKR1B10 [11,12,13]. AKR1B1 and AKR1B10 belong to the aldo-keto reductase superfamily, which includes about 190 cytosolic, NADPH-dependent monomeric enzymes [14,15]. The enzymes share approximately 71% amino acid sequence identity and show substrate and inhibitor specificities that largely overlap [16]. AKR1B1 catalyzes the reduction in a wide variety of carbonyl substrates [17,18]. It is particularly known for its ability to reduce glucose to sorbitol in the so-called polyol pathway, contributing to the onset of several secondary diabetes complications, including neuropathy, nephropathy, and retinopathy [19,20,21]. Besides its role in the polyol pathway, AKR1B1 acts as a detoxifying agent toward electrophilic by-products of lipid peroxidation, such as 4-Hydroxy-2-nonenal (HNE), which can compromise cell functions and induce cell death [22,23,24]. Additionally, evidence points to the involvement of AKR1B1 in inflammatory pathways. AKR1B1 converts the glutathione conjugate of HNE (GSHNE) to its corresponding reduced product, GSDHN, which in turn activates key regulatory factors of the inflammatory response, such as NF-κB and AP-1 [25,26]. In addition, AKR1B1 is involved in prostaglandin synthesis, as it shows PGF2α synthase activity and regulates Prostaglandin E2 (PGE2) production through the action of Prostaglandin F2 alpha (PGF2α) on its receptor [27]. The overexpression and increased activity of AKR1B1 have been reported in several tumour types, including rectal, ovarian, breast, liver, and cervical cancer [28].

Although the molecular mechanisms are not fully elucidated, emerging evidence suggests that AKR1B1 can contribute to cancer progression through its involvement in processes regulating inflammation, apoptosis, antioxidant responses, and chemoresistance [29]. Similar to AKR1B1, AKR1B10 is involved in lipid-derived carbonyl detoxification [30,31]. However, despite their similarities, they show markedly different kinetic properties in the presence of relevant physiologic substrates. Accordingly, AKR1B10 is not involved in the polyol pathway, but it emerges as a retinaldehyde and isoprenyl aldehyde reductase with much higher catalytic efficiency than AKR1B1 [32,33]. It is overexpressed in several types of tumours, including breast, liver, pancreas, and lung cancers [34,35,36,37,38]. Moreover, AKR1B10 is considered to be involved in different pro-tumorigenic mechanisms depending on the tumour type, including the activation of polycyclic aromatic hydrocarbon *trans*-dihydrodiols procarcinogens, the regulation of retinoic acid metabolism, the detoxification of cytotoxic aldehydes, the prenylation of Kras protein, and chemoresistance [36,39,40,41,42].

Due to this multiplicity of actions, these enzymes have been the focus of extensive drug development research. In the past decades, many efforts were made to develop inhibitors of AKR1B1, and several of them were investigated for their therapeutic potential. In vitro studies have demonstrated that AKR1B1 inhibitors effectively reduce intracellular sorbitol accumulation [19] and modulate pro-inflammatory signalling [19,43,44]. In addition, the efficacy of AKR1B1 inhibitors as potential drugs for the treatment of diabetic cardiomyopathy, nephropathy, and cataract was demonstrated in vivo [45,46,47]. However, the clinical translation of AKR1B10 inhibition represents a significant challenge, as several compounds have failed clinical trials due to side effects, poor bioavailability, or low efficacy [48]. Accordingly, to date, Epalrestat is the only inhibitor of AKR1B1 authorized for clinical use, and its sale is limited to Eastern countries. In this context, the ongoing phase III clinical trial evaluating AT-001, a novel synthetic inhibitor of AKR1B1, for the treatment of diabetic cardiomyopathy (NCT04083339) highlights the current and growing interest in targeting AKR1B1. Although research on AKR1B10 is less extensive than the decades of investigation devoted to AKR1B1, AKR1B10 has emerged as a promising target in cancer therapy, leading to several studies focused on the design and synthesis of selective inhibitors [49,50,51,52]. Evidence demonstrated that promising selective inhibitors of AKR1B10 enhance sensitivity to chemotherapeutic agents and suppress proliferation, migration, and metastatic potential of tumour cells [50].

Due to this multiplicity of actions, these enzymes have been the focus of extensive drug discovery efforts. Several natural compounds, including flavonoids, tannins, terpenoids, phenolics, and alkaloids, have been reported as AKR inhibitors [12,53,54,55,56,57]. This aligns with the growing interest in the characterization of the bioactive potential of plant-derived products, as it offers a double benefit: the biochemical and cytotoxic properties of many compounds are well-documented in the literature, while this approach promotes the valorisation of the natural source for its therapeutic potential. In this context, extracts of *Castanea mollissima* Blume (Chinese chestnut) have shown an inhibitory effect on AKR1B1, primarily associated with the presence of flavonoids and polyphenolic acids [4], thereby encouraging the research of AKR1B1/B10 in *Castanea sativa* species shells (CSSs).

CSS extracts are a rich source of compounds with antioxidant activity, a property that further enhances their biofunctional value [58]. However, the potential effects of compounds with AKR1B1/B10 inhibitory and antioxidant activities warrant careful consideration, as their impact may vary depending on the targets and the cellular context.

The dysregulation of AKR1B1 and AKR1B10 is implicated in pathological conditions in which several metabolic pathways are involved, including oxidative stress. AKR1B1 and AKR1B10 are both involved in reactions that overlap with oxidative stress pathways and may modulate cell response under oxidative conditions. Accordingly, studies have suggested that AKR1B10 may promote cancer cell survival [59] and chemoresistance [42] through several mechanisms, including the reduction in lipid-derived cytotoxic carbonyls. Similarly, as previously described, the ability of AKR1B1 to detoxify cytotoxic aldehydes is considered to modulate oxidative stress-related pathways, resulting in both cytoprotective and pro-inflammatory effects depending on the cell type and the specific aldehyde. A further consideration that needs to be made regards the role of the polyol pathway, which actively contributes to oxidative stress via NADPH depletion, imbalance of the NADH/NAD^+^ ratio, and advanced glycation end-product formation. Accordingly, molecules combining AKR1B1 inhibition with ROS-scavenging activity were proposed as a multitarget approach to delay or prevent cellular damage resulting from glucose toxicity [60]. Together, these functions illustrate the complex interplay between these enzymes and cellular redox homeostasis, suggesting that compounds that combine AKR1B1/B10 inhibitory activity with antioxidant properties may exert variable effects depending on the cellular environment. Therefore, the characterization of the antioxidant properties in addition to their AKR1B1/B10 inhibitory activity represents a preliminary step to assess the overall biofunctional potential of CSSs.

The possible modifications in the molecular landscape of these products during industrial peeling necessitate a thorough comparative evaluation of raw and processed chestnut shells. To date, no studies have reported the characterization and evaluation of extracts of industrially processed *Castanea sativa* species shells (CSSs). Here, we present a comparative characterization of the antioxidant activity of CSSs and industrial by-product extracts and the inhibitory effect on AKR1B1 and AKR1B10, with the aim of providing a base layer of knowledge to promote the rational recycling of this agricultural waste.

## 2. Results

### 2.1. Chemical Characterization and Radical Scavenging Capacity

First, we compared the amount of extractable materials from the two different samples, i.e., fresh (CSSs) and thermally processed (by-product) shells. A standard extraction procedure using a Soxhlet extractor was adopted. Additionally, two solvents (water or ethanol) were used. The extraction procedure lasted 6 h and was performed at the boiling point of the solvents (i.e., 78 °C for ethanol and 100 °C for water). In all cases, the extracting solution appeared colourless by the end of the process, suggesting that the extraction procedure was complete. The amount of extracted material from the four samples is reported in Table 1.

**Figure 1:**
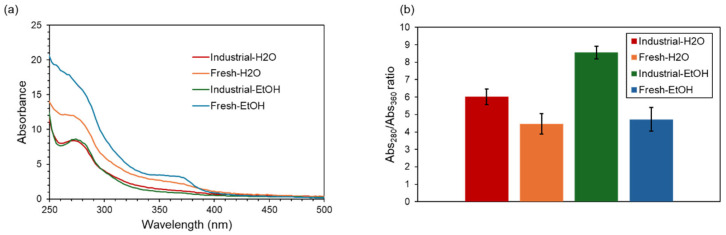
UV-VIS spectral analysis of the CSSs and industrial by-product extracts. (**a**) UV-VIS spectra of aqueous and ethanolic extracts of CSSs (orange and light blue lines, respectively) and of the industrial by-product (blue and green lines, respectively); (**b**) Intensity ratio of the absorbance at 280 nm and 360 nm of aqueous and ethanolic extracts of CSSs (orange and light blue bars, respectively) and of the industrial by-product (blue and green bars, respectively).

The UV-VIS spectra of the four extracts, normalized by the weight of dried extract, are reported in Figure 1a. Additionally, the intensity ratio between 280 and 360 nm absorbance shows a significant decrease of 1.5-fold on average, both in ethanolic and aqueous extracts, comparing fresh with industrial shells (Figure 1b).

Next, we turned our attention to the biochemical characterization of the extracts. In this view, we first assessed the total polyphenol index (TPI) and the oxygen radical absorbance capacity (ORAC). This last assay is a well-established protocol to measure the ROS scavenging capability. Results, shown in Table 1, indicate that the polyphenol index is higher in aqueous than in ethanolic extracts, with the greatest difference found in extracts from fresh shells. Extraction from processed shells, on the contrary, shows good efficiency also with the ethanolic extraction.

A more detailed characterization of the extracts was performed by LC-MS. To this end, an untargeted analysis was used, and the results were screened against public natural product libraries (GNPS) [61]. The library produced was finally cleaned for natural plant derivatives using the Plant Metabolome Hub [62]. After processing of the data, blank subtraction, and manual curation of the dataset with removal of artificial/synthetic structures, a total of 27 compounds quantified in at least one sample were tentatively assigned (see Table 2). Compound attributions are based on MS/MS spectral similarity and should be considered putative, with the possibility of spurious contributions from isomeric or structurally related analogues of the attributed compounds. Some tentatively assigned compounds correspond to structural classes reported in plants other than *Castanea*; these annotations likely reflect isomeric or structurally related analogues rather than the exact compounds listed and were retained only for class-level and functional-group analysis. Consequently, we aimed for a more hypothesis-driven attribution of functional groups in the extracts to gain a general overview of the contribution of different chemical traits rather than the specific identification of molecular candidates. From this perspective, categorization into functional classes suggests that both thermal treatment and the extraction solvent have a considerable impact on the chemical landscape of extractable products.

**Figure 2:**
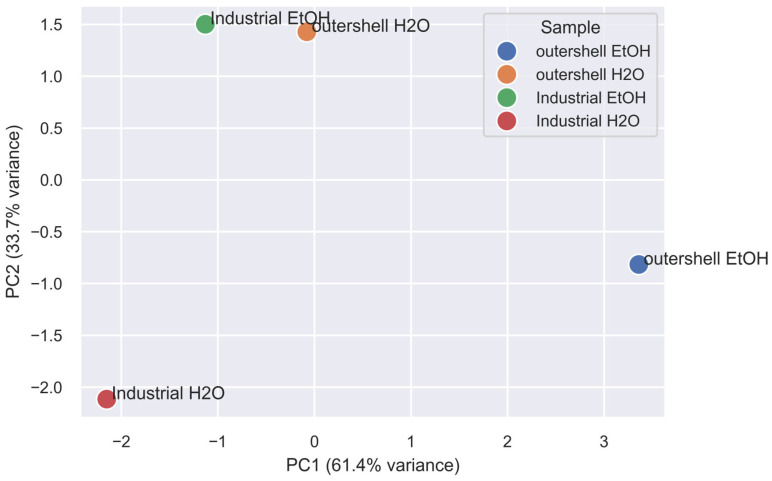
PCA based on molecules identified by LC-MS and quantified in the four extracts.

**Figure 3:**
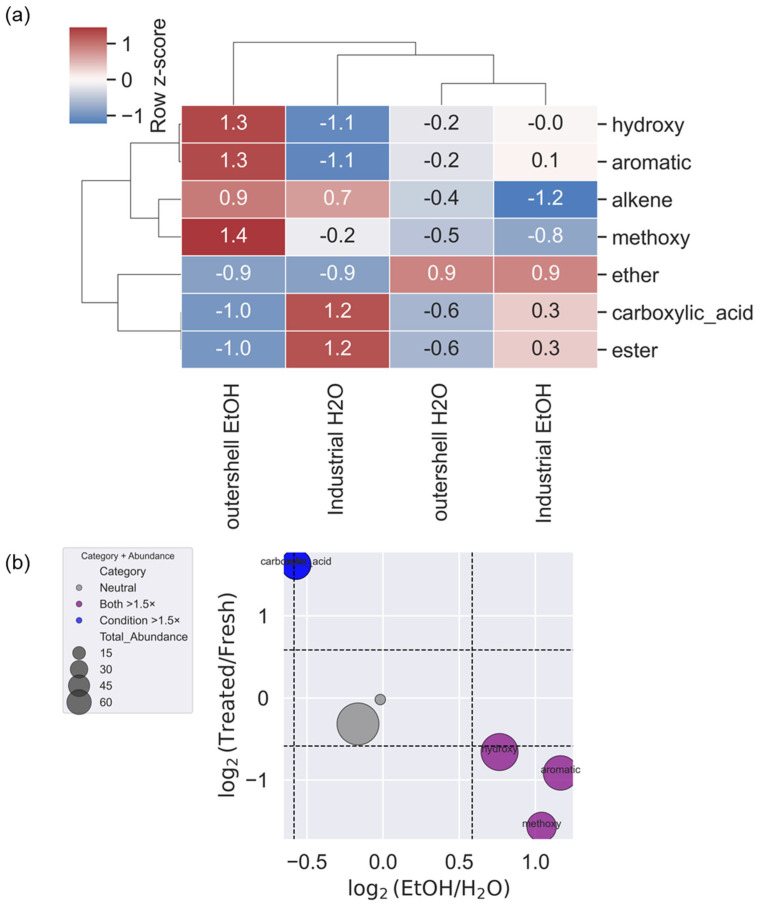
(**a**) Heatmap analysis based on categorization reported in Table S1 for the four extracts. (**b**) Categories of molecules mainly extracted in EtOH and H_2_O represented by the bubble importance graph.

The reference-matched compounds were categorized based on their chemical features (see Table S1), which involve both the presence of specific functional groups (e.g., hydroxyl, acidic, aromatic functional groups). The aim of this categorization was to derive some common chemical traits present in the extracts that might correlate with the observed antioxidant activity and with the inhibitory activity towards specific enzymes. This categorization was used to stratify the samples and processing procedures (Figure 2 and Figure 3).

### 2.2. Evaluation of the Extracts Asn Enzyme Inhibitors

Notably, some compounds attributed in the MS analyses (e.g., trihydroxyacetophenone, 2,4 dihydroxybenzoic acid methyl ester) are structurally related to compounds that were previously identified by our research group as effective inhibitors [57]. Thus, we further investigated the inhibitory potential of the extracts of the CSSs and the industrial by-products against AKR1B10 and AKR1B1, which were tested using HNE and L-Idose, respectively. The latter is an epimer of glucose, which is considered an excellent alternative to the physiological hexose due to its low K_M_ [63]. Several compounds identified or knowingly present in chestnut extracts, and, in particular, phenols, are known as PAINS (pan-assay interference compounds) and could potentially lead to protein aggregation and/or precipitation or chemically interfere with redox processes with NADPH/NADP+. Hence, a preliminary investigation was performed in each case to assess potential interference between the extract and NADPH. In all cases, the UV spectra of the solution did not change over time, indicating stability of the solution and negligible, if any, aspecific effect of the components on the enzyme or the cofactor. In the performed dose–response assays, all the extracts are effective in inhibiting the two enzymes, albeit with different efficiency. Inhibition curves and calculated IC50 values are reported in Figure S2 and Table 3, respectively.

An examination of the IC50 values evidences the clear discrepancy between the effectiveness of the ethanolic extract of the fresh product and the other samples. The IC50 value of this sample is higher (for both tested enzymes) by more than one order of magnitude compared to the best performing samples, and with a statistically significant difference (*p* < 0.05) from all the other samples, with the exception of the processed shell extracted in water. Notably, the ethanolic extract of fresh product shows the biggest difference in molecular composition when compared with the other extracts, although causality cannot be established at this stage due to the use of crude mixtures.

## 3. Discussion

In this study, we performed a comparative evaluation of raw and processed chestnut shells with a focus on the biochemical characterization of the antioxidant activity of CSSs and industrial by-product extracts and the inhibitory effect on AKR1B1 and AKR1B10. The amount of extracted material from the four samples is reported in Table 1 and clearly shows that the amount of extractable substances in processed shells is greater than what is obtainable from the unprocessed product.

This might be due to the lower amount of residual water in the processed samples before extraction or to an increased availability of small molecules linked to the degradation of cell walls during the industrial processing (approximately 30 min at 200–220 °C). The increased (1.5-fold) yield in extractables from fresh products when treated with water compared with ethanol can be explained by the higher ability of water to penetrate the untreated (and thus more compact) cell walls at boiling temperature compared to ethanol. Interestingly, the industrial desiccation treatment alters the ratio between water- and ethanol extractable substances, increasing the amount of substances soluble in ethanol and suggesting that chemical modification occurs to the extractables during the processing.

Another effect of the industrial processing can be hypothesized based on the shape of the measured UV-VIS spectra of the different samples (Figure 1a). It is obvious to observe that the fresh products have higher absorbance both at 280 and 360 nm compared with the dried ones. On the other hand, processed shells show almost superimposable UV-VIS spectra. This evidence is consistent with the observation that the chemical processes performed on the shells during industrial desiccation alter the relative abundance of aromatic (phenolic or flavonoid) compounds while increasing the extraction efficiency. A closer look at two distinctive absorption bands typical of these classes (i.e., 280 and 360 nm) highlights more pronounced alterations in the chemical landscape of the extractable products. When measuring the ratio between these two bands, which can be assumed to be linked to the phenolic/flavonoid ratio, it is evident that in both samples the ethanolic extraction increases the yield in phenolic compounds compared with the aqueous extraction (Figure 1b). The 360 nm absorption is usually associated with a typical band of cinnamoyl system in flavonoids, while the 280 nm band is related to transitions from the aromatic ring [64,65]. The observed alteration in the ratio between the bands suggests that thermal treatment is partially altering the cinnamoyl system of the flavonoid content and possibly inducing a degradation of glycosylated molecules, although a more detailed molecular characterization of the extracts is necessary to corroborate this hypothesis.

Regarding antioxidant capacity, in all cases, the 8–58 g of TPI/100 g observed is in line with previous reports [66]. Antioxidant capacity, measured by ORAC, shows a trend that is perfectly correlated with TPI in three extracts out of four, indicating a good relation between polyphenol content. In this sense, it is striking the difference observed in the ethanolic extract of processed shell, which indicated a much higher concentration of ORAC active compounds relative to the polyphenol amount. A PCA indicates (Figure 2) a striking difference in chemical composition of fresh outer shell extracted in ethanol compared with the other three extracts, with the smaller difference between fresh outer shell extracted in water and the ethanolic extract of processed shells. Interestingly, this is in line with the different behaviour both in ORAC/TPI and in enzyme inhibition assays.

When compound categories are analyzed, a clear distinction emerges between the extracts. As expected, less polar functional groups are mainly represented in ethanolic extracts (Figure 3b), while compounds with carboxylic acid as a functional group are more represented in aqueous extracts. Compounds bearing carboxylic acids are represented more frequently in treated samples, in line with the presumable thermal alteration caused by the processing. A closer examination of the single compounds evidences solvent- and treatment-specific distribution of several compounds of potential interest (Table 2 and Table S1). Interestingly, the ratio between the percentage of fatty acid derivatives in ethanol vs. water is nearly constant regardless of the processing status of the sample, suggesting good thermal stability of these extractables and a prominent role of the solvent in modulating the recovery. Cinnamic and benzoic acid derivatives are significantly decreased in processed shells, both in ethanol and aqueous extracts. This indicates a possible thermal degradation of these compounds upon thermal treatment. On the other side, there is a significant increase in compounds bearing carboxylic acid and ester functional groups, suggesting that thermally induced reactions on labile molecules might alter the molecular profile of the extracts. Overall, both thermal treatment and extraction solvent affect the quantity and the molecular properties, albeit with different effects that are related to both compound identity and presence of functional groups (Figure 3a,b). A closer analysis of the identified molecules provides some useful insights into the possible identification of antioxidant molecules that might explain the observed antioxidant effect. Seven identified molecules: 2,3-dimethoxybenzaldehyde [67], alpha,beta-dihydroresveratrol [68], 3,4-dihydroxybenzoate [69], tschimganidin, 2,4-dihydroxybenzoic-acid methyl ester [70], gingerol [71], and sambucinol with reported antioxidant activity. We then attempted to evaluate the correlation between the relative abundance of these seven identified antioxidants in the different samples and the measured radical scavenger activity measured by ORAC assay (Table 1). To this end, we calculated the intensity percentage of each compound in the processed samples, normalized by the total area of identified metabolites (Table S2). Next, we derived the overall percentage of the identified antioxidant compounds in each sample. Interestingly, we found a very strong positive correlation (Pearson coefficient 0.94) between the intensity percentage of the antioxidants in the extract and the measured ORAC activity. It is not possible to infer, from these data, a definitive correlation between identified compounds and antioxidant activity of the extract, but our data suggest that these candidates might be responsible for the biological activity observed.

Evaluation of the inhibitory activity of the extract was focused on AKR1B1 and AKR1B10 enzymes, which are involved in several pathological conditions. Given its role in pro-inflammatory and polyol pathways, AKR1B1 inhibition has long been explored as a strategy to prevent secondary diabetic complications. On the other hand, AKR1B10, a potential tumour marker, represents a target for suppressing tumour progression. Given their role as therapeutic targets, several compounds have been investigated as inhibitors of AKR1B1 and AKR1B10, despite the challenges related to bioavailability and selectivity [48,72,73]. Known inhibitors include synthetic, natural, or natural-derivative compounds.

The extracts of fresh outer shells and industrial by-products showed inhibitory activity with IC50 values in the range of 1.5–35 µg/mL against both enzymes (Figures S1 and S2, Table 3), and evidence a good inhibitory potential that might warrant additional evaluation in vitro.

Both the sample source and the solvent impacted the inhibitory potential against the two enzymes: the aqueous extract of fresh samples is the most effective on AKR1B1, while the ethanolic extract of the industrially processed shells is the most potent on AKR1B10. The variable degree of inhibitory activity of the extracts suggests that their different chemical compositions play a relevant role in modulating enzyme inhibition. Given that only four extract conditions were available, the resulting correlation coefficients must be interpreted with extreme caution and should be meant only as a guideline.

Future fractionation of the extracts, coupled with targeted metabolomics and direct testing of isolated fractions or pure compounds, will be required to clarify the contribution of these molecular classes to the overall inhibitory activity.

In this regard, an analysis was performed that correlates the chemical features of identified components with the inhibitory potential of the extracts. Of note, the presence of specific functional groups in inhibitors of either AKR1B1 or AKR1B10 is broadly reported (see, for example, the presence of phenolic functional groups in aldo-keto reductases [74,75]).

**Figure 4:**
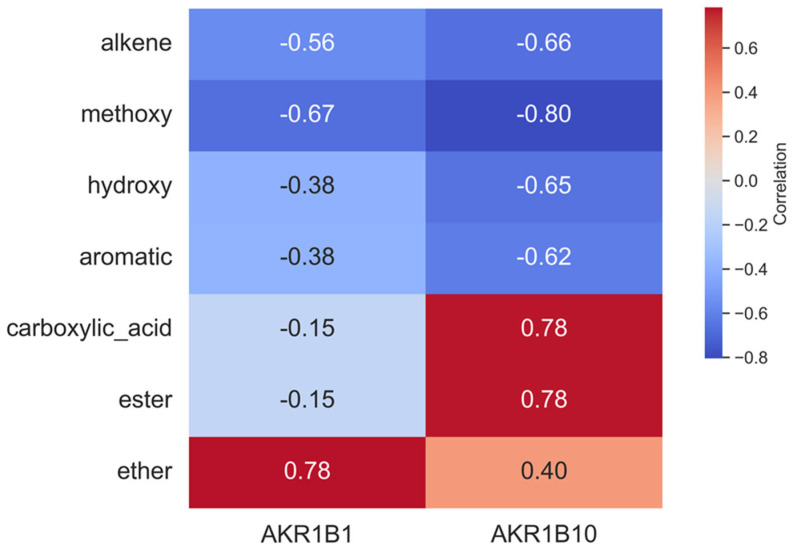
Correlation heatmap between the inhibition of the two target enzymes (enzyme 1: AKR1B1 and enzyme 2: AKR1B10) and the chemical classes identified in the extracts. Colour intensity reflects the strength and direction (positive or negative, correlated with class concentration) of the correlation.

The correlation analysis showed a different inhibitory pattern for the two enzymes (Figure 4). It is not trivial to infer a correlation between functional groups and inhibition of AKR1B1, although there is a correlation with the presence of ether functional groups, while the presence of ester and carboxylic acid functional groups correlates with lower IC50 values towards AKR1B10. Other chemical groups show a less defined trend, likely due to their presence in many compounds identified in the extracts, of which only a minor part is effective as an inhibitor. Although the expression of certain chemical classes does not fully align with the inhibitory properties reported in the literature, this effect can be attributed to the complex nature of the extract matrix. The bioactive compounds may exert inhibitory effects depending on their relative abundance, specific mechanisms of inhibition, and potential synergistic or antagonistic interactions. This correlation seems to highlight that some functional groups, which would naturally increase the concentration of a given substance in ethanolic or water extract, might contribute to the observed differences in that extract.

When a punctual analysis of the putatively identified compounds was performed, some structural classes emerged, such as coumarins and stilbenes, that have been reported as common traits of inhibitors of both AKRs [76,77,78]. It is not possible, based on the provided results, to establish a correlation between these motifs, which are present in several identified compounds, and the inhibition of one or both targets. However, this finding is consistent with the markedly different inhibitory potential and selectivity profiles of the molecules belonging to these classes. In this sense, the presence of gingerol among the identified compounds is a relevant example, since it has been reported for its anti-diabetes properties, including the inhibition of AKR1B1 reductase activity in vitro and in vivo [79].

Another interesting result that emerges from the analysis of identified structures is the presence of fatty acid derivatives that show a co-occurrence with stronger inhibition of both enzymes and a stronger effect on AKR1B10. Interestingly, fatty acids are inhibitors of AKR1B1 and AKR1B10, with a selective potential for the latter, depending on the level of unsaturation and the length of the chain [80].

Further analyses, including the fractionation of the extracts and more robust and sensitive quantification of target compounds, might clarify their relative, synergistic, or antagonistic contribution to the overall inhibitory activity observed in this study.

## 4. Conclusions

We provided a characterization of extracts of *Castanea sativa* shells, both fresh and industrially processed, as sources of bioactive mixtures with antioxidant and AKR1B1/AKR1B10 inhibitory activity. We showed that both industrial processing and extraction solvent impact the molecular landscape of extracts and that an appropriate choice of solvent can provide an extract with promising biologic activity, especially when antioxidant properties are concerned. These data support the concept of industrially processed chestnut shell–derived extracts as sources of molecularly complex extracts relevant for further investigation. These findings are promising from a circular economy perspective and promote further investigation of chestnut processing residues as renewable feedstock for the development of bioactive ingredients. In vitro studies are in progress to assess the true potential of these natural products.

## 5. Materials and Methods

### 5.1. Materials

Sodium chloride, Folin–Ciocalteu’s phenol reagent, 2,2′-Azobis(2-methylpropionamidine) dihydrochloride, 6-hydroxy-2,5,7,8-tetramethylchroman-2-carboxylic acid (Trolox), glycerol solution (83.5–89.5%), and EDTA were purchased from SIGMA-ALDRICH (St. Louis, MO, USA). D,L-dithiothreitol was purchased from MP Biomedicals, LLC (Illkirch, France). YM10 ultrafiltration membranes were obtained from Merck-Millipore (Darmstadt, Germany). DEAE Sepharose CL-6B and Blue Sepharose 6 Fast Flow were purchased from Cytyva (Uppsala, Sweden). Isopropyl-β-D-thiogalactopiranoside was purchased from Calbiochem^®^ (Merck KGaA, Darmstadt, Germany). L-Idose, gallic acid monohydrate, and Β-nicotinamide adenine dinucleotide phosphate reduced form, tetrasodium salt, were purchased from Biosynth (Nobelova, Bratislava, Slovakia). Sodium carbonate decahydrate, sodium dihydrogen phosphate, acetonitrile, ethanol, and formic acid were purchased from Carlo Erba Reagents (Milan, Italy). In addition, 96-well F-bottom black plates were purchased from Greiner Bio-One (Frickenhausen, Germany).

### 5.2. Sample Harvesting

CSSs were harvested from wild trees in Garfagnana, Tuscany, Italy, in October–November 2023. The outer shell was dissected from the inner shell and stored under vacuum at −20 °C until use. The industrial by-product was supplied by Azienda Agricola “Pieroni Anna Maria”, Pieve Toscana (LU), Italy. The by-product was obtained by fast (45 min) thermal treatment at 200–230 °C.

### 5.3. Sample Extraction

The ethanolic and aqueous extracts of both outer shells and by-products were obtained by placing five grams of sample with 100 mL of solvent in a Soxhlet extractor, which operated at the reflux temperature of the solvent (water or ethanol). The extraction process lasted for six hours. After the extraction had completed, the extract (80 mL) was allowed to cool at +4 °C overnight. The extract was then centrifuged at 10,000× *g* for 30 min at 4° C to remove the sediment and stored at −20 °C until use. The concentration of the extract was determined by weighing the residue after drying an aliquot of the extract in an evaporatory centrifuge (HyperVAC, VC124, Hanil Scientific Inc., Gimpo, Korea).

### 5.4. Total Polyphenol Index (TPI)

The Folin–Ciocalteu method was used to evaluate the TPI of the extract as previously reported [5], with minor modifications. Briefly, the extract was diluted in water to a final volume of 545 μL. Then, 50 μL of 2 N Folin–Ciocalteu’s phenol reagent and 405 μL of 20% (*w*/*v*) Na_2_CO_3_ solution were added. The mixture was incubated at 37 °C for 30 min. The absorbance was monitored at 765 nm using a Libra S35 PC spectrophotometer (Biochrom Ltd., Cambridge, UK). Gallic acid was used as a reference standard.

### 5.5. ORAC Assay

The ORAC assay is a method that measures the scavenging activity of a compound or mixture against peroxyl radicals, providing a direct measurement of the radical quenching effectiveness. The radical scavenging capacity of the extract was carried out according to what was previously reported [5], with minor modifications. Briefly, 25 μL of the extract, opportunistically diluted, was distributed in a 96-well F-bottom black plate. Then, 150 μL of 8 nM fluorescein solution prepared in 75 mM sodium phosphate buffer, pH 7.4 were added to the experimental wells. The plate was incubated at 37 °C for 30 min. The radical species were generated by the addition of 25 μL of 75 mM AAPH. The fluorescence was monitored kinetically in a Varioskan Flash (Thermo Scientific, Vantaa, Finland) plate reader at 37 °C (excitation/emission: 484/528 nm). Trolox (0–50 µM) was used to obtain a standard curve. Antioxidant activity was quantified by calculating the area under the curve, with results reported as mmol Trolox equivalents per gram of sample powder.

### 5.6. Enzyme Purification

The recombinant human AKR1B1 enzyme was produced and purified to achieve electrophoretic purity, as previously described [63]. The purified enzyme exhibited a specific activity of 5.3 U/mg. It was stored at −80 °C in a 10 mM sodium phosphate buffer (pH 7.0) supplemented with 2 mM DTT and 30% (*w*/*v*) glycerol.

The pET-30 vector, containing the sequence encoding human AKR1B10, was used to transform BL21 (DE3) pLysS *E. coli* cells. Protein expression was induced by the addition of 0.4 mM IPTG when the O.D. at 600 nm reached a value of 0.8. After overnight incubation at 30 °C, cells were collected by centrifugation and sonicated. Cell resuspension was centrifuged at 10,000× *g* for 30 min at 4 °C, and the soluble fraction, namely the crude extract, was collected. Purification of AKR1B10 consisted of two column chromatography steps. The crude extract was applied to a DEAE Sepharose CL-6B column (2.5 ø × 13 cm). The elution was performed at 20 mL/h in a 50 mM sodium phosphate pH 7 buffer supplemented with 2 mM DTT. Fractions showing AKR1B10 activity were pooled and dialysed with a YM10 ultrafiltration membrane until a final concentration of 0.2 mM DTT was reached. The pool of fractions was then applied to a Blue Sepharose 6 Fast Flow column (1.5 ø × 6 cm). The elution was performed at 20 mL/h in a 50 mM sodium phosphate pH 7 buffer. When the O.D. at 280 nm reached a baseline value, the elution of AKR1B10 was obtained by applying the standard buffer supplemented with 0.1 mM NADPH and 0.37 M NaCl. Fractions showing AKR1B10 activity were pooled and concentrated to a concentration of 2 mg/mL. The purified enzyme exhibited a specific activity of 1.7 U/mg. The enzyme purity was assessed by 12% SDS-PAGE electrophoresis, and the gel was stained with Coomassie Blu R. The enzyme was stored in 10 mM sodium phosphate buffer, pH 7, supplemented with 2 mM DTT and 30% (*v*/*v*) glycerol at −20 °C.

Prior to use, AKR1B1 and AKR1B10 underwent extensive dialysis against 10 mM sodium phosphate buffer at pH 7.0, which was supplemented with 0.5 mM DTT for AKR1B10 dialysis.

### 5.7. Enzyme Activity Assay

The activity of the recombinant enzymes was determined at 37 °C, monitoring the decrease in absorbance at 340 nm linked to NADPH oxidation (ε_340_ = 6.22 mM^−1^ cm^−1^) using a Libra S35 PC spectrophotometer (Biochrom Ltd., Cambridge, UK). The standard assay mixture of AKR1B1 (0.7 mL) contained 0.25 M sodium phosphate buffer, pH 6.8, 0.18 mM NADPH, 0.4 M ammonium sulphate, 0.5 mM EDTA, and 4.7 mM D, L-glyceraldehyde. The assay mixture of AKR1B10 contained 100 mM sodium phosphate buffer, pH 7.0, 0.18 mM NADPH, and 15 mM D, L-glyceraldehyde. The same conditions were applied for the inhibition assays in the presence of L-Idose and HNE as substrates. For the ethanolic extract assays, the mixtures contained 1.4% (*v*/*v*) of ethanol, which does not significantly affect the activity of the enzymes. Before adding the substrate to start the reaction, a blank assay was performed to correct for the autooxidation of NADPH and to monitor the possible phenol-catalyzed oxidation of the reduced cofactor. The controls included all the assay reagents except for the sample. The inhibitory ability of the extracts was calculated by the following equation:

$$Residual\;activity\;\%=\frac{(\Delta Abs/\min_{sample}-\Delta Abs/\min_{blank})\;}{\left(\Delta Abs/\min_{control}-\Delta Abs/\min_{blank\;}\right)}*100$$

where $\Delta Abs/\min_{sample},\;\Delta Abs/\min_{blank},\;\Delta Abs/\min_{control}$ represent the rate of change in absorbance per minute of the sample, the blank, and the control, respectively.

IC50 values were determined by the non-linear regression analysis using GraphPad Prism version 8.0 (GraphPad Software, San Diego, CA, USA). For each value, the 95% confidence limits (CLs) are reported.

### 5.8. LC-MS Analysis

HPLC–Orbitrap–MS/MS analyses were conducted on an Orbitrap Exploris 120 mass spectrometer (Thermo Scientific, Bremen, Germany) coupled to a Vanquish HPLC system (Thermo Scientific, Germering, Germany). Chromatographic separation was achieved using a Jupiter^®^ Proteo column (0.3 mm × 150 mm, 4 µm; Phenomenex, Danaher group, Torrance, CA, USA). The mobile phases consisted of water with 0.1% formic acid (phase A) and acetonitrile (phase B). The gradient elution program was as follows: 5–100% B from 0 to 45 min, isocratic at 100% B from 45 to 60 min, followed by re-equilibration to initial conditions from 60 to 100 min. The flow rate was set at 0.005 mL/min, the column temperature at 30 °C, and the injection volume at 5 µL.

Chestnut samples, diluted 1:10 in 0.1% formic acid, were analyzed in positive electrospray ionization mode over a mass range of m/z 100–800. The ion spray voltage was set to 4000 V, with sheath gas at 5 (arb), auxiliary gas at 1 (arb), and the ion transfer tube temperature at 320 °C. Data-dependent acquisition was performed using automatic dynamic exclusion, selecting only singly charged precursor ions, with four MS/MS events acquired per full MS scan. Data acquisition was carried out using Thermo Scientific™ Xcalibur™ 4.7 software.

### 5.9. Metabolomic Data Processing

Untargeted metabolomic profiles of chestnut peel extracts were processed using MS-DIAL (version 4.9.221218) [81]. Raw LC–MS/MS data files were imported and subjected to peak detection and alignment using the default centroiding and smoothing parameters. The minimum peak height, mass tolerance for MS1 and MS2 features, and retention time tolerance were set to 10,000 counts, 0.003 Da, and 4 min, respectively. Metabolite annotation was performed by matching MS/MS spectra against the publicly available GNPS spectral library [61] using an identification score cut-off of 85%. Putative candidate molecules were filtered based on their presence in the blank sample and manually curated to remove spurious synthetic compounds. The peak area of selected candidates was normalized based on the measured weight of dry substance in each extract. The final table was exported for downstream statistical analyses. Molecular abundance data were processed using a custom Python-based pipeline (Python version: 3.13.9), starting from a manually curated dataset (Table S1) containing metabolite identifiers, quantitative sample intensities, and chemical class annotations. Abundances were normalized to enable comparison of relative molecular contributions across samples. Multivariate analyses were performed on autoscaled data (zero mean, unit variance), including principal component analysis (PCA). Differential molecular patterns were assessed through log_2_ fold-change calculations between experimental conditions, and statistical significance was estimated using Welch’s *t*-test. Correlations between individual molecule abundances and bioactivity parameters (expressed as 1/IC50) were evaluated using Pearson correlation coefficients. The Python pipeline is available upon request to the authors.

### 5.10. Other Methods

Protein concentration was assessed using the Coomassie Brilliant Blue staining method [82], using bovine serum albumin as a standard. Synthesis of HNE was performed by the Department of Chemistry and Industrial Chemistry, University of Pisa, as previously reported [83]. The compound was synthesized as a diethyl acetal and stored in hexane at −80 °C. The corresponding free aldehyde was obtained by removing the solvent through evaporation and adding 1 mM HCl. After 30 min of incubation at 4 °C, the concentration of the corresponding aldehyde was evaluated by spectrophotometric measurement (ε_224nm_ = 13.75 mM^−1^ cm^−1^).

## Figures and Tables

**Table 1:** Summary of the amount of the extracted material, TPI (g GAE/100 g ± SEM), and radical scavenging capacity assays (mmol Trolox Equivalent/g ± SEM) of the extracts of CSSs and industrial by-product.

	Extraction Yield	TPI ^1^	ORAC Assay
	mg/g of Crude Product	(GAE/100 g) ^2^	(mmol Trolox Equivalent/g)
aqueous CSSs	103	57.7 ± 3.9 a^i,ii^	4.49 ± 0.29 a,c^v^
ethanolic CSSs	73	8.5 ± 2 b	0.84 ± 0.21 b^vi^
aqueous by-product	164	42 ± 2 a^iii,iv^	3.08 ± 0.041 b,c^vii^
ethanolic by-product	213	26 ± 1.6 b	8.71 ± 0.97 a

^1^ Total polyphenol index evaluated by Folin–Ciocalteu’s method; ^2^ GAE: Gallic acid equivalent; data are expressed as mean values ± SEM. Different letters denote statistically significant differences. ^i^: *p*-value < 0.0001 compared to ethanolic CSSs; ^ii^: *p*-value < 0.001 compared to ethanolic by-product; ^iii^: *p*-value < 0.01 compared to ethanolic CSSs; ^iv^: *p*-value < 0.05 compared to ethanolic by-product; ^v^: *p*-value < 0.01 compared to ethanolic CSSs; ^vi^: *p*-value < 0.0001 compared to ethanolic CSSs; ^vii^: *p*-value < 0.01 compared to ethanolic by-product. Kruskal–Wallis test with Dunn’s post hoc correction, n ≥ 4.

**Table 2:** LC-MS tentative identification of plant metabolites in the extracts.

ID	RT	MS1	Metabolite Name
1	-	166.17	2,3-dimethoxybenzaldehyde
2	0.99	161.06	aminoadipic acid
3	1.34	175.12	arginine
4	1.36	149.05	methionine
5	2.7	245.07	uridine
6	2.7	182.08	L-tyrosine
7	3.39	217.1	tschimganidin
8	3.39	169.05	2,4-dihydroxybenzoic acid, methyl ester
9	3.7	288.15	piperanine
10	3.8	252.12	N-acetyl-L-tyrosine ethyl ester
11	3.82	205.09	tryptophan
12	3.99	245.18	isoleucylisoleucine
13	4.35	185.07	asperlactone
14	4.4	176.06	guanidinosuccinic acid
15	4.47	231.08	alpha,beta-dihydroresveratrol
16	4.83	153.01	3,4-dihydroxybenzoate
17	5.48	203.05	psicose
18	6.49	267.15	sambucinol
19	6.92	168.07	carbazole
20	7.1	169.05	2,4,6-trihydroxyacetophenone
21	9.11	330.2	hetisine
22	13.43	191.06	7-methoxy-4-methylcoumarin
23	39.04	171.09	yohimbinic acid monohydrate
24	39.24	341.33	sucrose
25	54.88	351.5	gingerol
26	57.2	217.1	decarestrictine
27	80.27	338.34	erucamide

**Table 3:** IC50 values of CSSs and industrial by-product extracts on AKR1B1 and AKR1B10.

	AKR1B1			AKR1B10		
	IC50 (µg/mL)	CI 95%	R^2^	IC50 (µg/mL)	CI 95%	R^2^
aqueous CSSs	1.5	1.233–1.928	0.9711	6.2	4.553–8.758	0.9412
ethanolic CSSs	35 ^a^	28.24–42.38	0.964	21 ^a^	15.36–29.25	0.9282
aqueous by-product	5.4	3.857–7.531	0.9277	3.3	2.254–4.868	0.8952
ethanolic by-product	3.6	2.569–5.048	0.9428	2.5	1.725–3.501	0.9483

Data are expressed as mean values with CI (95%). IC50 (μg/mL), 50% inhibitory concentration; CI, confidence interval; R^2^, coefficient of determination of the regression model. ^a^ Statistically significant difference (*p* < 0.05) from aqueous and ethanolic by-products.

## Data Availability

The raw datasets presented in this article are not readily available because they are part of an ongoing study. Requests to access the datasets should be directed to Giovanni Signore. The dataset derived from raw data and used to generate the results is reported in the Supplementary Materials. The custom code used to process these results is available at https://github.com/giovannisignore/MetabolitesPipeline/blob/main/PipelineMetabolites.py, accessed on 16 January 2026.

## Supplementary Materials

The following supporting information can be downloaded at: https://www.mdpi.com/article/10.3390/molecules31030563/s1, Figure S1: Inhibition curves of the extracts against AKR1B1; Figure S2: Inhibition curves of the extracts against AKR1B10; Table S1: List of putatively identified compounds, areas, and functional group attributions; Table S2: TIC-normalized percentages (based on MS intensity) of the putatively identified compounds.

## Abbreviations

The following abbreviations are used in this manuscript:

AAPH	2,2′-Azobis(2-methylpropionamidine) dihydrochloride
AP-1	Activator protein-1
AKR1B1	Aldo-keto reductase family 1, member B1, also known as Aldose Reductase
AKR1B10	Aldo-keto reductase family 1, member B10
CSSs	*Castanea sativa* shells
DTT	Dithiothreitol
EDTA	Ethylenediaminetetraacetic acid
FAO	Food and Agriculture Organization
GNPS	Global Natural Products Social Molecular Networking
GSDHN	glutahionyl-1,4-dihydroxynonane
GSHNE	3-glutathionyl-4-hydroxynonane
HNE	4-hydroxy-2,3-nonenal
IPTG	Isopropyl-β-D-tiogalattopiranoside
LC-MS	Liquid Chromatography–Mass Spectrometry
NF-κB	Nuclear Factor kappa-light-chain-enhancer of activated B cells
ORAC	Oxygen radical absorbance capacity
PGF2α	Prostaglandin F2 alpha
PGE2	Prostaglandin E2
PCA	Principal component analysis
ROS	Reactive oxygen species
TPI	Total polyphenol index
