# Supplementary material for: Exploring Castanea sativa Shells (CSSs) as a Source of AKR1B1 and AKR1B10 Inhibitors: From Extraction to Bioactivity Testing
Source: Molecules. 2026 Feb 5;31(3):563. doi: 10.3390/molecules31030563 (PMC12899490; doi:10.3390/molecules31030563)
Supplement: Supplementary file 1 [file molecules-31-00563-s001.zip › molecules-4091592-supplementary.pdf]

Supplementary information

Exploring Castanea sativa Shells (CSSs) as a Source of AKR1B1 and AKR1B10 Inhibitors: From Extraction to Bioactivity Testing

Supplementary Table S1: List of putatively identified compounds, areas, and functional groups attribution

Metabolite name	outershell EtOH	outershell H2O	Industrial EtOH	Industrial H2O	aromatic	hydroxy	methoxy	alkene	carboxylic_acid	ester	ether
2,3-dimethoxybenzaldehyde	83.16915		4004.525	502.99							
2,4,6-Trihydroxyacetophenone		13446.37	190838		X	X					
Sambucinol				214.8643		X					
Decarestrictine			8542.397					X	X	X	
Asperlactone		1304.053	690.1993			X	X				
7-methoxy-4-methylcoumarin	1354.707			51.41741			X	X	X	X	
Benzoic-acid,-2,4-dihydroxy-,-methyl-ester	0.002882	1057.95	1639.647	0.00469		X	X	X			X
Carbazole					X						
Arginine									X	X	
Tryptophan	10699.3	3063.948	115433.4	3936.178				X	X	X	
Erucamide		10415.7						X			
Guanidinosuccinic-acid			47368.01						X	X	
Gingerol			711.3506	1162.042		X	X	X			
Methionine									X		
Hetisine						X					
alpha,beta-Dihydroresveratrol	1550.904	337.3128	1733.006	191.6441		X	X	X			
Isoleucylisoleucine						X					
Psicose						X					
3,4-Dihydroxybenzoate	737.4203	4938.534		3027.978				X	X	X	
Piperanine	78427.24	2736.355	1044.993	1787.128	X	X	X	X			
Uridine	59.39824	91.79423	4793.872	129.1936	X	X					
Sucrose						X	X				
Tschimganidin					X		X		X	X	
L-Tyrosine					X				X	X	
Yohimbinic-acid-monohydrate			1564.513		X	X					
N-Acetyl-L-tyrosine-ethyl-ester		851.4996	17897.49			X	X	X			X
Aminoadipic acid									X	X	

Supplementary Table S2: TIC-normalized percentages (based on MS intensity) of the putatively identified compounds

Metabolite name	outershell EtOH	outershell H2O	Industrial EtOH	Industrial H2O
2,3-dimethoxybenzaldehyde	0.089514		1.010577	4.571207
2,4,6-Trihydroxyacetophenone		35.15987	48.15962	
Sambucinol				1.952701
Decarestrictine			2.155748	
Asperlactone		3.409867	0.174178	
7-methoxy-4-methylcoumarin	1.458052			0.467285
Benzoic-acid,-2,4-dihydroxy-,-methyl-ester	3.1E-06	2.766351	0.413779	4.26E-05
Carbazole				
Arginine				
Tryptophan	11.5155	8.01168	29.13062	35.77225
Erucamide		27.2352		
Guanidinosuccinic-acid			11.95373	
Gingerol			0.179515	10.56072
Methionine				
Hetisine				
alpha,beta-Dihydroresveratrol	1.669216	0.882013	0.437339	1.741674
Isoleucylisoleucine				
Psicose				
3,4-Dihydroxybenzoate	0.793675	12.91339		27.51847
Piperanine	84.41011	7.155082	0.263713	16.24154
Uridine	0.063929	0.240026	1.209775	1.17412
Sucrose				
Tschimganidin				
L-Tyrosine				
Yohimbinic-acid-monohydrate			0.394818	
N-Acetyl-L-tyrosine-ethyl-ester		2.22652	4.516587	
Aminoadipic acid				

Figure S1 Inhibition curves of the extracts against AKR1B1

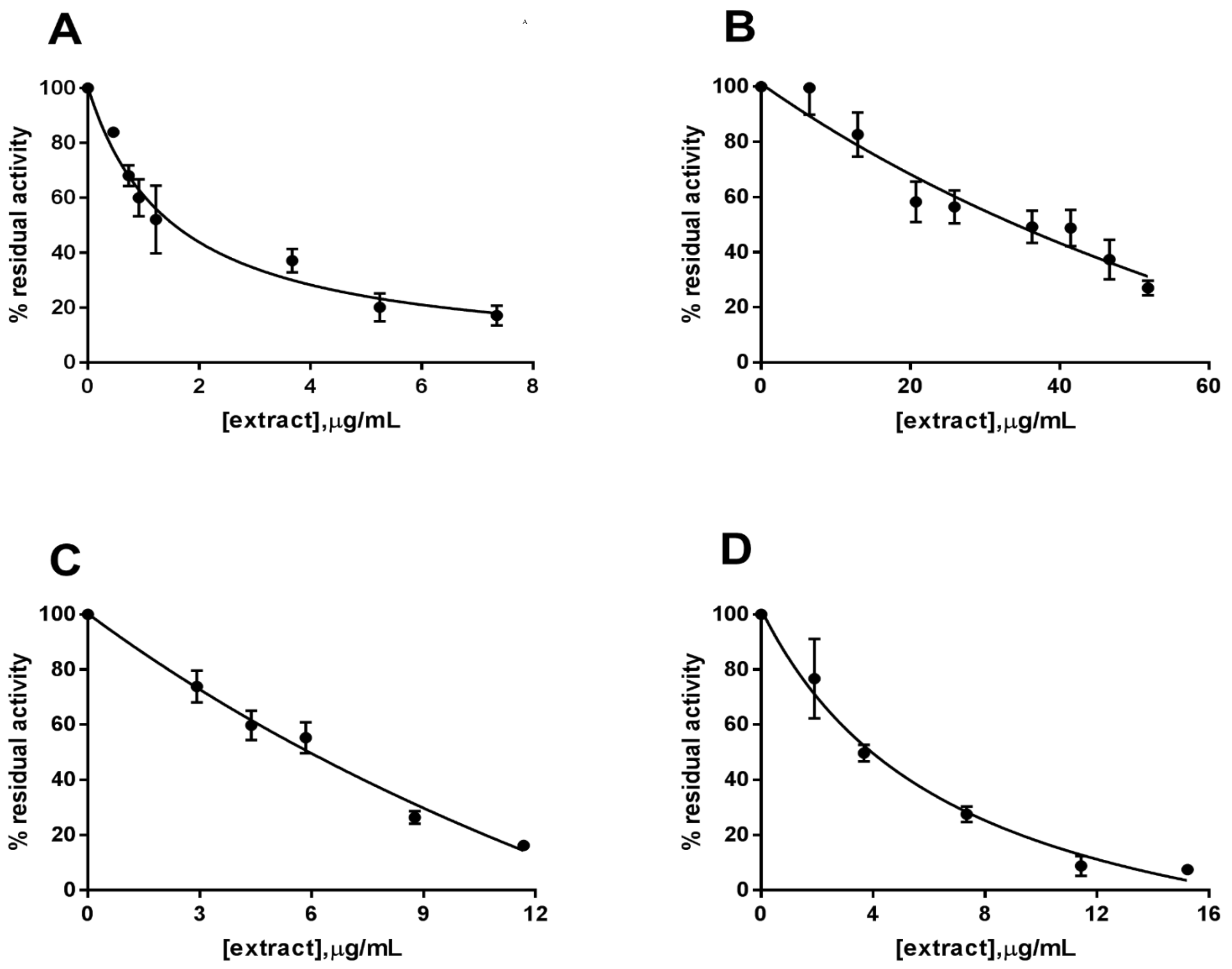

Figure S 1 Residual activity % of AKR1B1 (10 mU) in the presence of 1 mM L-Idose as function of extract concentration: aqueous CSS (panel A), ethanolic CSS (panel B), aqueous by-product (panel C), and ethanolic by-product (panel D). Results are presented as mean ±standard deviation of three independent measurements

Figure S2 Inhibition curves of the extracts against AKR1B10

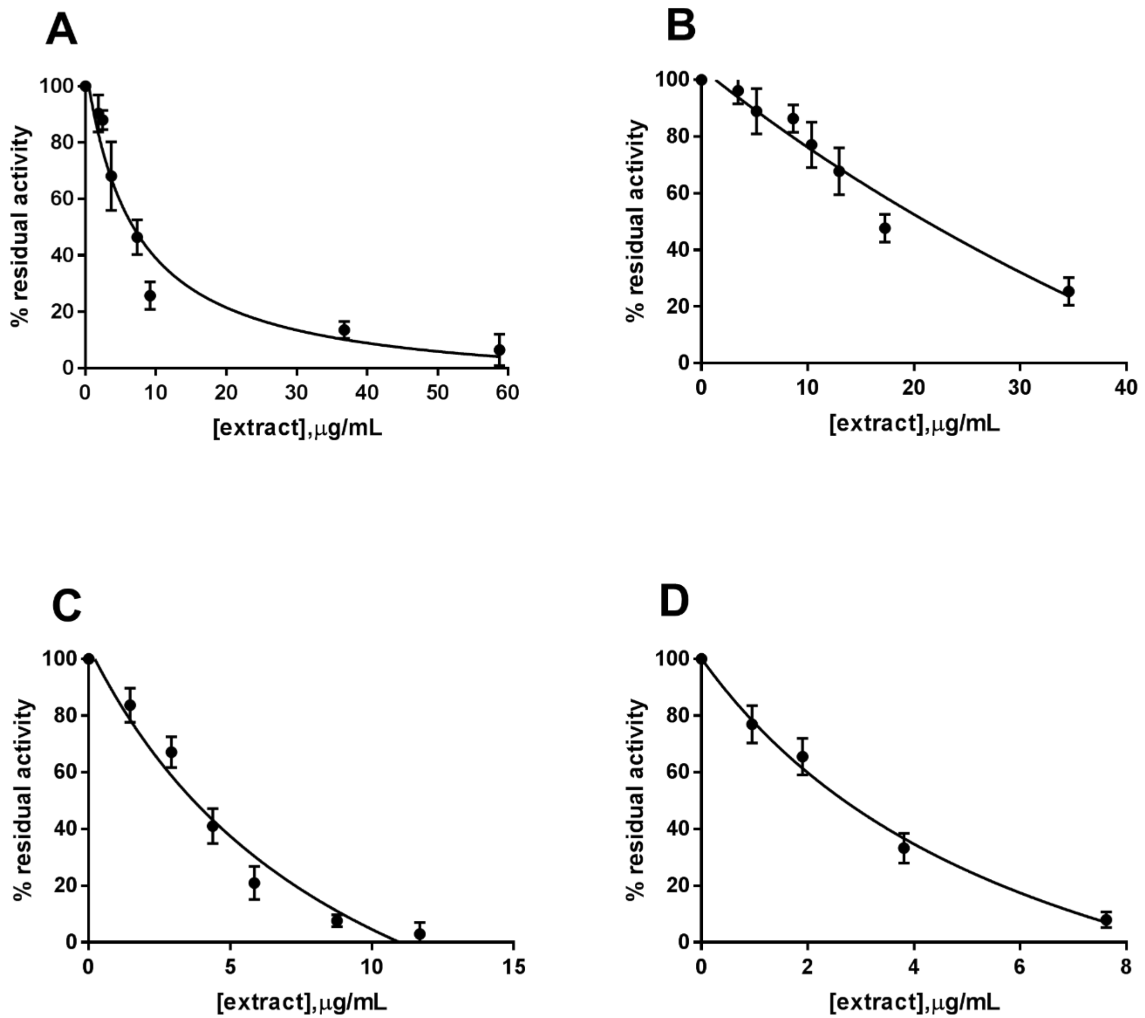

Figure S 2 Residual activity % of AKR1B10 (5 mU) in the presence of 0.04 mM HNE as function of extract concentration: aqueous CSS (panel A), ethanolic CSS (panel B), aqueous by-product (panel C), and ethanolic by-product (panel D). Results are presented as mean ± deviations of three independent measurements.
